# Theoretical Analysis of Efficiency of Multi-Layer Core-Shell Stationary Phases in the High Performance Liquid Chromatography of Large Biomolecules

**DOI:** 10.3390/molecules24152849

**Published:** 2019-08-06

**Authors:** Szabolcs Horváth, Fabrice Gritti, Róbert Kormány, Krisztián Horváth

**Affiliations:** 1 Department of Analytical Chemistry, University of Pannonia, Egyetem utca 10, H-8200 Veszprém, Hungary; 2 Waters Corporation, 34 Maple Street, Milford, MA 01757, USA; 3 Egis Pharmaceuticals PLC, Keresztúri út 30-38, 1106 Budapest, Hungary

**Keywords:** multi-layer core-shell particles, chromatographic efficiency, resolution, general rate model, moment analysis

## Abstract

Modern analytical applications of liquid chromatography require columns with higher and higher efficiencies. In this work, the general rate model (GRM) of chromatography is used for the analysis of the efficiency of core-shell phases having two porous layers with different structures and/or surface chemistries. The solution of the GRM in the Laplace domain allows for the calculation of moments of elution curves (retention time and peak width), which are used for the analysis of the efficiency of bi-layer particles with and without a non-porous core. The results demonstrate that bi-layer structures can offer higher separation power than that of the two layers alone if the inner layer has smaller surface coverage (retentivity) and the pore size and pore diffusion of the outer layer is either equal to or higher than that of the inner layer. Even in the case of core-shell phases, there is an increase in resolution by applying the bi-layer structure; however, we can always find a mono-layer core-shell particle structure with a larger core size that provides better resolution. At the optimal core size, the resolution cannot be further improved by applying a bi-layer structure. However, in case of the most widely produced general-purpose core-shell particles, where the core is ∼70% of the particle diameter, a 15–20% gain of resolution can be obtained by using well-designed and optimized bi-layer core-shell phases.

## 1. Introduction

Higher separation efficiency and faster speed have always been of great interest in high performance liquid chromatography (HPLC) [1,2,3]. The diameter of HPLC particles has been shrinking through the years, so that sub-2 μm totally porous particles are now used widely for separating small molecules. Columns of superficially porous particles (SPP) [4,5] have shown even further efficiency advantages, such that some users prefer these over totally porous particles (TPP). Recent studies have reported both the advantages and disadvantages of columns packed with core-shell and totally porous sub-2 μm particles [6,7].

Using very fine particles (sub-2 and sub-1 μm), due to the narrow peaks, sensitivity and separation are improved at the cost of pressure. Knox and Saleem were the first to discuss the compromise between speed and efficiency [8]. A critical aspect is the effect of frictional heating at ultra high pressure, causing temperature gradients within the columns [9]. The radial temperature gradient, due to the heat dissipation at the column wall, can cause significant losses in plate count [10]. Gritti et al. concluded that both longitudinal and radial temperature gradients are more significant when the column length is decreased [11]. In some practical situations, the disadvantages of using columns of sub-2 μm particles outweigh the advantages, particularly for routine analyses involving less technology-based personnel [6].

Shell particles manifest the advantages of porous and non-porous particles. The concept of superficial, or shell stationary, phase was introduced by Horváth et al. in the late 1960s [12,13]. Fused-core packing materials are commercially available in different diameters (5 μm, 2.7 μm, 2.6 μm, and 1.6–1.7 μm) [14]. The actual advantages of columns packed with these new core-shell particles lie in the diminution of both the longitudinal diffusion B coefficient (−20 to −30%) and the eddy dispersion A term (−40%). The decrease of the B coefficient was expected, because a significant fraction of the column volume (20%) is now occupied by non-porous silica through which analytes cannot axially diffuse. The C term of shell particles is also more favorable than that of the fully porous particles, especially for large molecules (proteins); however, the benefits of core-shell particles mostly lie in the A and B terms in separation of small molecules. It is well-known that larger SPPs (e.g., 2.5–2.7 μm) provide almost the same separation efficiency and resolution as sub-2 μm totally porous particles, but at one-half to one-third of the operating pressure [15,16].

Core-shell particles have proved to be equal to or to surpass the resolution and the efficiency of fully-porous particles of smaller sizes and monolith columns, not only in terms of being height equivalent to a theoretical plate, but also in overall kinetic performance [17]. The huge number of papers describing applications that take advantage of core-shell particle columns have demonstrated that the pace of adoption of this technology is growing exponentially among practitioners in virtually all fields of analytical chemistry [18,19].

It has been shown that the unique structure of superficially porous particles provides significant advantages for the separation of different types of compounds, provided that the analysis be carried out carefully, knowing the consequences of the different options. Resolution analysis has showed that the separation power of superficially porous particles increases with decreasing shell thickness if the strength of the eluent is decreased to compensate for the retention change caused by the decreased surface area of the stationary phase. This work showed also that, although columns packed with superficially porous particles can be used with conventional HPLC systems, the width of the bands eluting from them is narrower than that of peaks eluted from columns packed with fully porous particles. As a consequence, the extra-column volume of the chromatograph used must be significantly minimized to avoid losing the separation power provided by these new, unique, and efficient packing materials [20].

Gritti et al. [21] measured the mass transfer kinetics and the performance of columns packed with particles having similar but different and original structures (non-porous, superficially porous with one or two concentric shells, and fully porous) using small molecules (uracil and naphthalene) and large proteins (insulin, lysozyme, and BSA). The columns used were packed with the fully porous particles 2.5 μm Luna-C 18 100 Å, core-shell particles 2.6 μm Kinetex-C 18 100 Å, 3.6 μm Aeris Widepore-C 18 200 Å, and prototype 2.7 μm multi-layer core-shell particles (made of two concentric porous shells with 100 and 300 Å average pore size, respectively), and with 3.3 μm non-porous silica particles. The results demonstrated that the porous particle structure and solid–liquid mass transfer resistance had practically no effect on the column efficiency for small molecules. In contrast, for proteins, this third contribution to the height equivalent to a theoretical plate, hence the porous particle structure, together with eddy dispersion, governed the kinetic performance of the columns. Mass transfer kinetics of proteins were observed to be fastest for columns packed with core-shell particles having either a large core-to-particle ratio or having a second, external, shell made of a thin porous layer with large mesopores (200–300 Å) and a high porosity (≃0.5–0.7). The structure of this external shell seems to speed up the penetration of proteins into the particles.

The aim of this paper is to investigate, by a theoretical approach, whether higher efficiency could be achieved by using multi-layer core-shell particles.

## 2. Theory

### 2.1. Structure of Multi-Layer Core-Shell Particles

**Figure 1:**
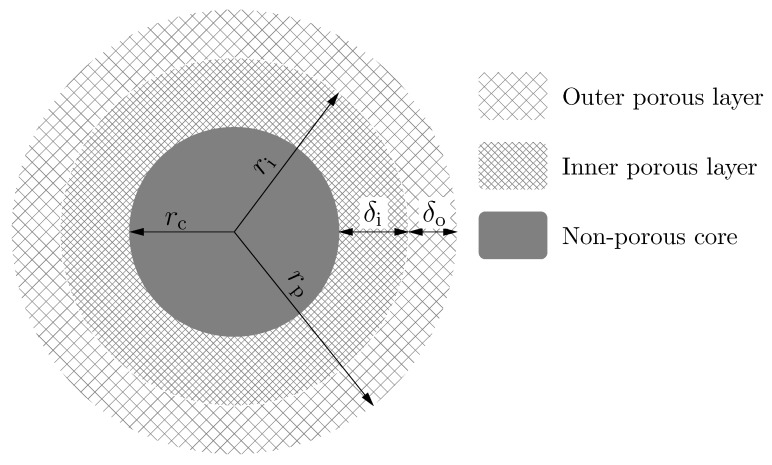
Structure of the bi-layer core-shell particle.

Multi-layer core-shell particles consist of three different parts (see Figure 1).

1. A non-porous core with a radius $r_{c}$:
  (1) $$r_{c}=\rho \;r_{p},$$
  where $r_{p}$ is the radius of particle (Figure 1) and ρ is the factor of proportonality between the radius of the inner solid core and the radius of the particle. This region is impermeable to the compounds analyzed and to the molecules of eluent. Note that, if ρ is equal to 0, there is no solid core inside the particle, while, in the case of ρ=1, the whole particle is non-porous, such as the Kovasil phases [22]. Accordingly, 0≤ρ≤1.
2. A porous inner layer with a thickness $\delta_{i}$"
  (2) $$\delta_{i}=r_{i}-r_{c}=\left(\beta -\rho \right)\;r_{p},$$
  where $r_{i}$ is the radius of the outer surface of the inner porous layer (Figure 1) and β is the factor of proportonality between $r_{i}$ and $r_{p}$. This layer has a given porosity ($\varepsilon_{i}$) and surface chemistry. Note that, if β is equal to ρ or 1, the particle has only one porous layer. If ρ≤β≤1, the particle has two porous layers.
3. A porous outer layer with a tickness $\delta_{o}$:
  (3) $$\delta_{o}=r_{p}-r_{i}=\left(1-\beta \right)r_{p}.$$
  Depending on the manufacturer, this layer may or may not have different porosity ($\varepsilon_{o}$) and surface chemistry than the inner porous layer.

The porosity of the shells ($\varepsilon_{p}$) depends on the thickness of the different layers, and the size of the non porous core:

(4) $$\varepsilon_{p}=\varepsilon_{o}\left(1-\beta^{3}\right)+\varepsilon_{i}\left(\beta^{3}-\rho^{3}\right).$$

The total porosity of the column packed with multi-layer core-shell particles can be calculated as

(5) $$\varepsilon_{T}=\varepsilon_{e}+\left(1-\varepsilon_{e}\right)\left[\varepsilon_{o}\left(1-\beta^{3}\right)+\varepsilon_{i}\left(\beta^{3}-\rho^{3}\right)\right]=\varepsilon_{e}+\left(1-\varepsilon_{e}\right)\varepsilon_{p},$$

where $\varepsilon_{e}$ is the external porosity of the column.

### 2.2. General Rate Model for Multi-Layer Core-Shell Particles

Many processes take place in the column, during the migration of solute molecules, which cause band broadening. There is no single rate-controlling process. The general rate model of chromatography considers the axial dispersion as the sum of axial and eddy dispersion, the external film mass transfer resistance, the intraparticle diffusion (including the pore and surface diffusions), and the rate of adsorption–desorption. As the processes taking place inside and outside a particle are considered separately in the GR model, two mass balance equations for the solute have to be written, one for the interstitial flowing mobile phase, and one for the stagnant mobile phase inside the particles.

For the processes in the interstitial liquid phase, the following mass balance equation can be written:

(6) $$\frac{\partial \;c_{e}\left[z,t\right]}{\partial \;t}+u_{e}\frac{\partial \;c_{e}\left[z,t\right]}{\partial \;z}+F\frac{\partial \;\bar{q}\left[z,t\right]}{\partial \;t}=D_{L}\frac{\partial^{2}\;c_{e}\left[z,t\right]}{\partial \;z^{2}},$$

where $c_{e}$ is the concentration of the solute in the interstitial volume; $u_{e}$ is the interstitial velocity of the eluent; $D_{L}$ is the axial dispersion coefficient defined for the external mobile phase, which is the sum of the molecular and the eddy diffusion coefficients; *F* is the phase ratio, defined as $\frac{1-\varepsilon_{e}}{\varepsilon_{e}}$; and $\bar{q}$ is the value of the stationary phase concentration, *q*, averaged over the entire particle. For a spherical particle, it can be calculated as

(7) $$\bar{q}=\frac{3}{r_{p}^{3}}\int_{M}^{r_{p}}r^{2}\;q\;dr.$$

The derivative in the third term of Equation (6) is the rate of adsorption averaged over the particle. It is calculated as

(8) $$\frac{\partial \;\bar{q}\left[z,t\right]}{\partial \;t}=\frac{3}{r_{p}}\;k_{f}\;\left(c_{e}\left[z,t\right]-c_{o}\left[r_{p},t\right]\right),$$

where $k_{f}$ is the external mass transfer coefficient and $c_{o}\left[r_{p},t\right]$ the concentration of the solute within the pores at the surface of the particle.

For the processes taking place inside of the multi-layer core-shell particle, two differential equations have to be written: One for the outer porous layer, and another for the inner porous layer. Accordingly,

(9) $$A_{o}\frac{\partial \;c_{o}\left[r,t\right]}{\partial \;t}=D_{o}\left(\frac{\partial^{2}\;c_{o}\left[r,t\right]}{\partial \;r^{2}}+\frac{2}{r}\frac{\partial \;c_{o}\left[r,t\right]}{\partial \;r}\right),$$

(10) $$A_{i}\frac{\partial \;c_{i}\left[r,t\right]}{\partial \;t}=D_{i}\left(\frac{\partial^{2}\;c_{i}\left[r,t\right]}{\partial \;r^{2}}+\frac{2}{r}\frac{\partial \;c_{i}\left[r,t\right]}{\partial \;r}\right),$$

where the superscripts *i* and *o* represent the inner and outer porous layers, respectively; $c_{o}$ and $c_{i}$ are the concentrations of the solute in the stagnant mobile phase of pores in the two layers; $D_{o}$ and $D_{i}$ are the diffusion coefficients of the solute in particle pores; and

(11) $$A_{o}=\varepsilon_{o}+\left(1-\varepsilon_{o}\right)\;K_{o},$$

(12) $$A_{i}=\varepsilon_{i}+\left(1-\varepsilon_{i}\right)\;K_{i},$$

where $K_{o}$ and $K_{i}$ are the Henry coefficients of the solutes in the two layers. In the equations above, an infinitely fast adsorption–desorption kinetics was assumed (q[r]=Kc[r]). Note that, even if the retention of solutes is described here with simple equilibrium parameters (one for each layer), the kinetics of adsorption of solutes can be significantly more complex than this simple approach [23,24]. It was shown, in [24], that several thousands of similar but slightly different kinetic processes are lumped together in the adsorption of a similar-sized peptide as that used here as a model.

The following boundary condition was used at the outer surface of the particle:

(13) $$D_{o}{\left.\frac{\partial \;c_{o}\left[r,t\right]}{\partial \;r}\right|}_{r=rp}=k_{f}\left(c_{e}\left[z,t\right]-c_{o}\left[r_{p},t\right]\right).$$

At the boundary between the two porous layers, an infinitely fast mass transfer was assumed. Accordingly:

(14) $$c_{i}\left[r_{i},t\right]=c_{o}\left[r_{i},t\right]$$

and

(15) $$D_{i}{\left.\frac{\partial \;c_{i}\left[r,t\right]}{\partial \;r}\right|}_{r=r_{i}}=D_{o}{\left.\frac{\partial \;c_{o}\left[r,t\right]}{\partial \;r}\right|}_{r=r_{i}}.$$

There is no mass flux through the porous core. Accordingly, the boundary condition at the surface of the non porous core can be written as

(16) $${\left.\frac{\partial \;c_{i}\left[r,t\right]}{\partial \;r}\right|}_{r=r_{c}}=0.$$

For the sake of simplicity, a simple dirac delta injection was assumed as initial condition.

(17) $$c_{e}\left[0,t\right]=\delta \left(t\right).$$

### 2.3. Height Equivalent to a Theoretical Plate of Chromatographic Columns

The performance of different chromatographic columns is conveniently compared on the basis of the values of their height equivalent to a theoretical plate (HETP), which can be calculated from the statistical moments of the peak eluted:

(18) $$\mathrm{HETP}=\frac{\mu_{2}^{\prime }}{\mu_{1}^{2}}L,$$

where $\mu_{1}$ is the first absolute, $\mu_{2}^{\prime }$ the second centralized moment of the peak, and *L* the column length.

A closed-form analytical solution of the system of partial differential equations defined in the previous section [Equations (6)–(17)] is impossible to derive in the time domain. Although the solution in the Laplace domain can be derived, it cannot be transformed back into the time domain. However, the moments of the peaks can be calculated from the Laplace transform of the solution easily [25,26,27]:

(19) $$\mu_{c}={\left(-1\right)}^{n}\frac{\partial^{n}\;\log C\left(s,L\right)}{\partial s^{n}},$$

where C(s,L) denotes the Laplace transform of the elution profile at the outlet of the column (*L*), *s* is the Laplace variable, and n>0.

## 3. Methods

The separation power of the multi-layer particles was investigated for a typical large peptide (e.g., insulin: Molecular weight, M, ∼6 kDa; molecular diffusion coefficient, $D_{m}$, $∼6\times 10^{-5}\;\frac{{\mathrm{cm}}^{2}}{\min}$) by Equations (20)–(31) which was derived from Equations (6)–(19) using Mathematica 10.0 (Wolfram Research Inc., Champaign, IL, USA). The numerical calculations were carried out using software written in-house in Python programming language (v. 3.6, Anaconda Python Distribution), using the NumPy and SciPy packages. The values of the numerical parameters necessary for the numerical calculations, such as the column parameters, the particle size, and so on, are listed in Table 1.

## 4. Results and Discussions

### 4.1. General Solution of the GR Model

The first moment of the general rate model [Equations (6)–(17)]—in other words, the retention time—is given by

(20) $$\mu_{1}=\frac{L}{u_{e}}\left(1+k_{1}\right),$$

where

(21) $$k_{1}=k_{o}+k_{i}$$

and

(22) $$k_{o}=F\left(1-\beta^{3}\right)A_{o},$$

(23) $$k_{i}=F\left(\beta^{3}-\rho^{3}\right)A_{i}.$$

The second moment is given by

(24) $$\mu_{2}=\frac{2\;L}{u_{e}}\left(\delta_{ax}+\delta_{f}+\delta_{d}\right),$$

where $\delta_{ax}$, $\delta_{f}$, and $\delta_{d}$ are the contributions of the axial dispersion, external film mass transfer, and intra-particle diffusion to the variance of peak eluted, respectively.

(25) $$\delta_{ax}=\frac{D_{L}}{u_{e}^{2}}{\left(1+k_{1}\right)}^{2}$$

(26) $$\delta_{f}=\frac{r_{p}}{3\;k_{f}\;F}\;k_{1}^{2}$$

(27) $$\begin{array}{c} \delta_{d}=\frac{r_{p}^{2}}{15\;F}\left[\frac{k_{i}^{2}}{D_{i}\;\beta }\frac{\beta^{6}-5\beta^{3}\rho^{3}+9\beta \rho^{5}-5\rho^{6}}{{\left(\beta^{3}-\rho^{3}\right)}^{2}}+\frac{1-\beta }{D_{o}\;\beta }\right.\\ \left.\left(\frac{k_{o}^{2}\;\beta \left(5\beta^{3}+6\beta^{2}+3\beta +1\right)+5\;k_{i}\;k_{o}\;\beta \left(2\beta^{3}+3\beta^{2}+3\beta +1\right)+5k_{i}^{2}\left(\beta^{2}+\beta +1\right)}{{\left(\beta^{2}+\beta +1\right)}^{2}}\right).\right] \end{array}$$

From Equation (18), it follows that the HETP of a column packed with multi-layer core-shell particles is given by

(28) $$\mathrm{HETP}=\frac{2\;u}{{\left(1+k_{1}\right)}^{2}}\left(\delta_{ax}+\delta_{f}+\delta_{d}\right)=h_{ax}+h_{f}+h_{d},$$

where $h_{ax}$, $h_{f}$, and $h_{d}$ are the contributions of the axial dispersion, external film mass transfer, and intra-particle diffusion to the HETP, respectively.

The apparent retention factor, *k*, of a compound can be calculated from Equation (20), considering that the hold-up time of the column, $t_{M}$, is equal to the first statistical moment of the band of a non-retained compound. Accordingly,

(29) $$k=\frac{t_{R}-t_{M}}{t_{M}}=F\;\frac{K_{o}\left(1-\beta^{3}\right)+K_{i}\left(\beta^{3}-\rho^{3}\right)}{1+F\;\varepsilon_{p}}.$$

Equations (20)–(28) can be used as a general basis for the calculations of moments or HETP of columns packed with particles having different structures. For example, if β=1 or β=ρ, the particle behaves as a core-shell particle; $k_{1}$ and $\delta_{d}$ become

(30) $$k_{1}=F\;\left(1-\rho^{3}\right)A_{o},$$

(31) $$\delta_{d}=\frac{r_{p}^{2}}{15\;D_{o}}\frac{k_{1}^{2}}{F}\;\frac{1+2\rho +3\rho^{2}-\rho^{3}-5\rho^{4}}{{\left(\rho^{2}+\rho +1\right)}^{2}},$$

which is identical to the results obtained previously for superficially porous particles.

### 4.2. Separation Efficiency of Bi-Layer Fully Porous Particles

For the illustration of the separation efficiency of a superficially porous bi-layer particle, the HETP curves were plotted against the beta coefficient. The parameters of a typical large peptide (insulin) were used as the model molecule for our calculations. In respect of the surface coverage of the two porous layers and the diffusion constant of the molecules in the pores, 12 HETP curves could be plotted. The parameters presented in Table 2 were used to plot these curves.

Retentions in the inner and outer layers depend on the values of $A_{i}$ and $A_{o}$, which can be calculated by Equations (11) and (12), respectively. Their values depend on the porosity (ε) and surface coverage (Henry coefficient, *K*) of the layer. The latter refers to the number of $C_{18}$ groups bonded to the surface of the given layer. The $A_{i}$ and $A_{o}$ values used in the calculations were 1.2 and 3, equivalent to retention factors, *k*, of 0.75 and 2.45 in the case of traditional totally porous particles, respectively. Besides the molecular diffusion coefficient of the solute, the values of pore diffusion parameters, $D_{o}$ and $D_{i}$, depend mainly on the ratio of particle diameter to the nominal pore diameter. Accordingly, $10^{-5}$ and $10^{-6}\frac{{\mathrm{cm}}^{2}}{\min}$ pore diffusion coefficients correspond to pore sizes of 300 and 100 Å, respectively, in the case of a typical large peptide.

**Figure 2:**
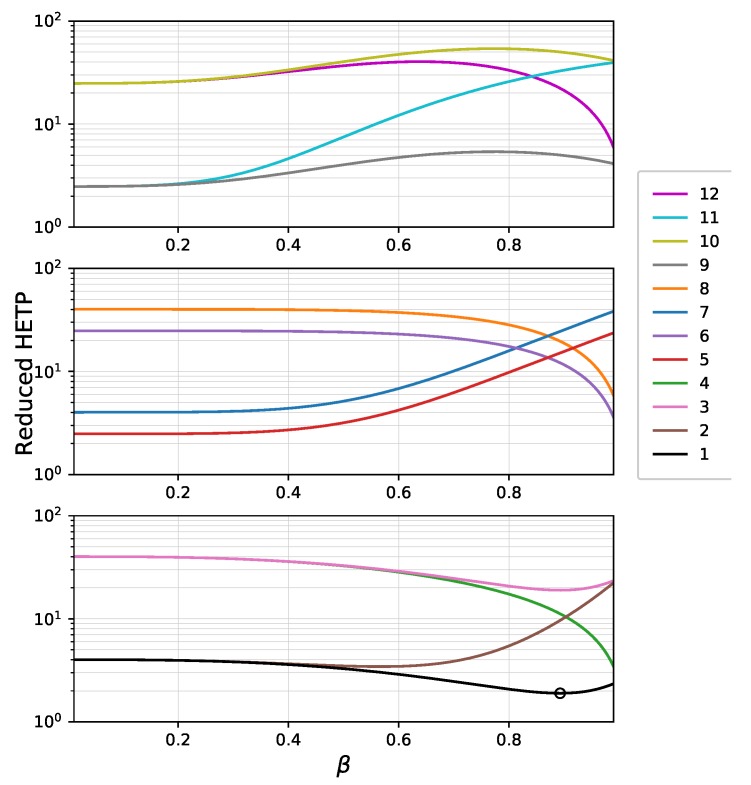
Reduced height equivalent to a theoretical plate (HETP) of totally porous bi-layer particles plotted against β for the scenarios presented in Table 2, where β is the factor of proportionality between the radius of the outer surface of the inner porous layer and the particle radius (see Figure 1 and Equations (2) and (3)).

Figure 2 shows the calculated HETP of totally porous (ρ=0) bi-layer particles as a function of factor of proportionality between the radius of the outer surface of the inner porous layer, $r_{i}$, and the particle radius, $r_{p}$ ($\beta =r_{i}/r_{p}$, as shown in Figure 1). The thickness of the inner and outer layers can be calculated by Equations (2) and (3) with ρ=0. In order to demonstrate the effect of the bi-layer structure on the chromatographic efficiency, the contributions of axial and film mass transfer processes ($\delta_{ax}$ and $\delta_{f}$) were neglected. Accordingly, only $\delta_{d}$ was taken into account during the calculations (see Equations (25)–(28)). Note, however, that for large peptides, pore diffusion is the most significant contribution of mass transfer processes to the overall HETP [25]. The calculated results correlate well with measured reduced HETPs on conventional and wide-pore core-shell phases [28,29]. Figure 2 shows that the structure of a bi-layer stationary phase affects its efficiency significantly. For a mono-layer totally porous phase, the best efficiency is given with A=3 and $D_{p}=10^{-5}\;\frac{{\mathrm{cm}}^{2}}{\min}$ as could be expected. In that case, the reduced HETP is ∼2.3. Depending on the surface retentivity and pore diffusion of the inner and outer layers, the efficiency may increase or decrease as the ratio of porous layers changes. In general, it can be concluded that the efficiency increases as the ratio of the larger pore size layer increases, as it could be expected. In the cases of scenarios 5, 7, and 11, the efficiency decreases monotonically, while in cases of scenarios 4, 6, and 8, it increases monotonically as the inner layer increases. The HETP lines of scenarios 1, 2, 3, 9, 10, and 12 have either local minima (1, 2, 3) or maxima (9, 10, 12). The former cases reveal that it is possible to combine two different porous layers resulting in a higher efficiency than that of the two layers alone. The common factors in scenarios 1, 2, and 3 are that (1) the inner layer has smaller surface coverage ($A_{i}=1.2$ versus $A_{o}=3$) and (2) the pore size and pore diffusion of the outer layer is either equal to or higher than that of the inner layer. The highest efficiency was obtained in case of scenario 1 (black line, minimum point marked with a circle), where both layers had faster pore diffusion and the inner layer had a smaller retention than the outer one.

### 4.3. Separation Efficiency of Bi-Layer Core-Shell Particles

**Figure 3:**
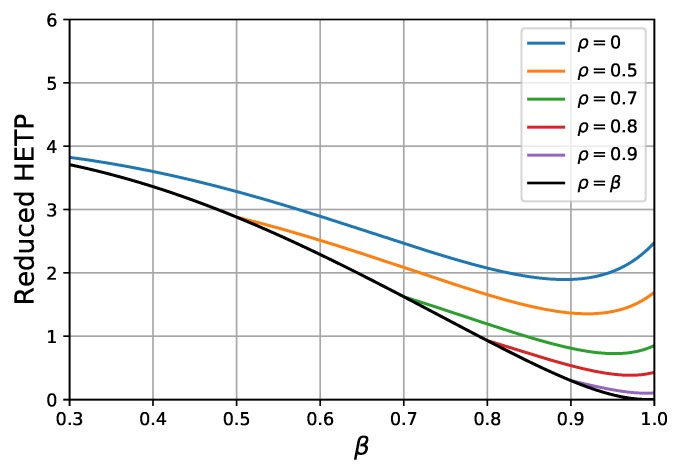
Reduced HETP plotted against the β at different values of ρ.

In Figure 3 the reduced HETPs of bi-layer core-shell phases can be seen against β at different core sizes. The black covering line (β=ρ, see Figure 1 and Equations (2) and (3)) corresponds to mono-layer particles. The best combination of the parameters from the previous section (scenario 1) was used to study the separation efficiency of the core-shell bi-layer phases. Close examination of Figure 3 highlights that, at any given core size, the efficiency can be further increased by applying a bi-layer structure. At the same time, however, we can always find a mono-layer core-shell structure which offers higher efficiency than any bi-layer solid core particle. Note, however, that the decreasing shell thickness causes a decrease of the surface area of the stationary phase, resulting in a decrease of retention and eventually of the actual resolution between the compounds when the porous layer becomes too thin.

**Figure 4:**
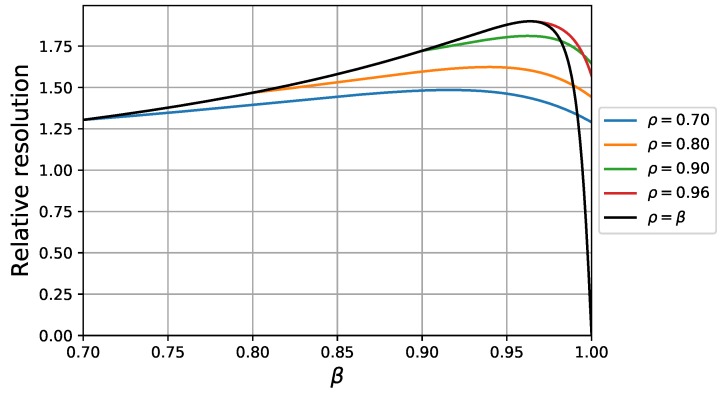
Relative resolution plotted against β at different values of ρ.

In chromatographic practice, resolution is a more important parameter than HETP. If we wish to study the resolution capability, we have to define a new compound. The $A_{o}$ and $A_{i}$ parameters of the new compound was multiplied with 1.2. In other words, the selectivity of the two compounds was 1.2. In Figure 4, the calculated resolution curves are plotted against β at different values of ρ. Similarly to the HETP curves shown in Figure 3, we can see that there is an increase in resolution by applying bi-layer superficially porous particles; however, up to a certain core size, we can always find a mono-layer core-shell particle structure with a larger core size that provides a better resolution. Further increase of the core size above a certain optimal value, however, decreases the chromatographic resolution, due to the significant decrease of retention. At the optimal core size, the resolution cannot be further improved by applying a bi-layer structure, since the less retentive inner layer decreases the retention time difference of the two compounds. Accordingly, the best separation power can be obtained by optimizing the core size of a mono-layer core-shell particle. It is important to note, however, that the optimal core diameter depends significantly on the type of analyte, as was shown in [20]. Economically, it is not feasible to design and optimize too many specific stationary phases. The core sizes of core-shell phases optimized for analysis of large macromolecules are typically 90–95% of the particle diameter (note that Figure 4 also suggests this core size). Our results clearly demonstrate that there is not any advantage of using a bi-layer structure for those phases. In the case of smaller core sizes, however, the advantages of a bi-layer structure are more significant. The size of core in the most-widely produced general purpose core-shell particles is ∼70% of the particle diameter (ρ=0.7). As Figure 4 suggests, a 15–20% gain of resolution can be obtained by using well-designed and optimized bi-layer core-shell phases.

## 5. Conclusions

As was shown in [20], the general rate model is a powerful tool for designing and optimizing high-performance stationary phase particles, as it considers all the processes that take place in the flowing interstitial mobile phase and in the stagnant liquid phase inside the particles. In this work, the general rate model was used for the analysis of efficiency of bi-layer stationary phases with and without non-porous cores. The solution of GRM in the Laplace domain allowed for analysis of the efficiency of these phases. The results suggest that, by careful design and optimization, bi-layer structures can offer higher separation power than mono-layer phases if the outer layer has larger retentivity and pore diffusion than the inner layer. The results also demonstrated that resolutions cannot be further improved by a bi-layer structure if the size of non-porous core is optimized for the given separation. If the goal, however, is to develop a stationary phase for general purposes, a 15–20% gain of column efficiency can be reached by using a bi-layer structure.

The derived equations can also be used for the design of more exotic stationary phases, such as hollow particles that contain holes (eluent) at the center of the particles, allowing for fast diffusion and fast mass transfer. Even if the production of these exotic stationary phases is not feasibly technically at present, it may be interesting to theoretically analyze the applicability of such phases. It is important to note, however, that the approach introduced in this work cannot predict the mechanical stability of particles, nor the wall effect during column packing that influences the applicability and the efficiency of the column significantly. In spite of these limitations, the application of the general rate model can accelerate the design and optimization of novel stationary phases.

## Figures and Tables

**Table 1:** The values of the numerical parameters necessary for the numerical calculations.

Parameter	Value
Column length (*L*)	10 cm
Column diameter ($d_{c}$)	0.3 cm
Particle diameter ($d_{p}$)	2.7 μm
External porosity ($\varepsilon_{e}$)	0.4
Interstitial mobile phase velocity ($u_{e}$)	5 $\frac{\mathrm{cm}}{\min}$

**Table 2:** Retention ($A_{i}$ and $A_{o}$, see. Equations (11) and (12)) and pore diffusion parameters ($D_{i}$ and $D_{o}$) of the inner and outer layers used for the calculation of separation efficiencies of superficially porous bi-layer particles.

No.	$A_{i}$	$A_{o}$	$D_{i}$	$D_{o}$
			$\frac{{\mathrm{cm}}^{2}}{\min}$	$\frac{{\mathrm{cm}}^{2}}{\min}$
1	1.2	3	$10^{-5}$	$10^{-5}$
2	1.2	3	$10^{-6}$	$10^{-5}$
3	1.2	3	$10^{-6}$	$10^{-6}$
4	1.2	3	$10^{-5}$	$10^{-6}$
5	1.2	1.2	$10^{-6}$	$10^{-5}$
6	1.2	1.2	$10^{-5}$	$10^{-6}$
7	3	3	$10^{-6}$	$10^{-5}$
8	3	3	$10^{-5}$	$10^{-6}$
9	3	1.2	$10^{-5}$	$10^{-5}$
10	3	1.2	$10^{-6}$	$10^{-6}$
11	3	1.2	$10^{-6}$	$10^{-5}$
12	3	1.2	$10^{-5}$	$10^{-6}$

## Abbreviations

The following abbreviations and symbols are used in this manuscript:

MDPI	Multidisciplinary Digital Publishing Institute
DOAJ	Directory of open access journals
HPLC	High pressure liquid chromatography
HETP	Height equivalent to a theoretical plate
GRM	General rate model
SPP	Superficially porous particle
TPP	Totally porous particle
$r_{p}$	particle radius
$r_{c}$	core radius
$r_{i}$	radius of the outer surface of the inner porous layer
ρ	factor of proportonality between the $r_{c}$ and $r_{p}$
β	factor of proportonality between $r_{i}$ and $r_{p}$
$\delta_{i}$	thickness of the inner porous layer
$\delta_{o}$	thickness of the outer porous layer
$\varepsilon_{p}$	total porosity of the porous shells
$\varepsilon_{i}$	porosity of the inner porous layer
$\varepsilon_{o}$	porosity of the outer porous layer
$\varepsilon_{e}$	external porosity of the column
$\varepsilon_{T}$	total porosity of the column
$u_{e}$	interstitial velocity of the eluent
*F*	phase ratio
$D_{L}$	axial dispersion coefficient
$c_{e}\left[z,t\right]$	concentration of the solute in the interstitial volume
$c_{o}\left[r_{p},t\right]$	concentration of the solute within the pores at the outer perimeter of the particle
*q*	concentration of solute adsorbed on the surface of stationary phase
$\bar{q}$	*q* averaged over the entire particle
$k_{f}$	external mass transfer coefficient
$c_{i}$	concentration of the solute in the stagnant mobile phase of pores in the inner layer
$c_{o}$	concentration of the solute in the stagnant mobile phase of pores in the outer layer
$D_{i}$	pore diffusion coefficient of solute in the inner layer
$D_{o}$	pore diffusion coefficient of solute in the outer layer
$K_{i}$	Henry coefficient of the solute in the inner layer
$K_{o}$	Henry coefficient of the solute in the outer layer
$\mu_{1}$	first normalized moment of the peak
$\mu_{2}^{\prime }$	second centralized moment of the peak
*L*	column length
C(s,L)	Laplace transform of the elution profile at the outlet of the column
$d_{c}$	column diameter
$d_{p}$	particle diameter
$\delta_{ax}$	contribution of axial dispersion to the variance of peak eluted
$\delta_{f}$	contribution of external film mass transfer to the variance of peak eluted
$\delta_{d}$	contribution of intra-particle diffusion to the variance of peak eluted
$h_{ax}$	contribution of axial dispersion to the HETP
$h_{f}$	contribution of external film mass transfer to the HETP
$h_{d}$	contribution of intra-particle diffusion to the HETP
$k_{i}$	zone retention coefficient of the inner layer
$k_{o}$	zone retention coefficient of the outer layer
$k_{1}$	zone retention coefficient, sum of $k_{i}$ and $k_{o}$
$A_{i}$	retention parameter of the outer layer
$A_{o}$	retention parmeter of the outer layer
*k*	apparent retention factor
$t_{R}$	retention time
$t_{M}$	hold-up time of the column
*z*	spatial variable
*s*	Laplace variable
*r*	radial variable
*t*	time
